# The Genetic Basis of the Polycystic Ovary Syndrome: A Literature Review Including Discussion of *PPAR-γ*

**DOI:** 10.1155/2007/49109

**Published:** 2007-02-25

**Authors:** Ugur Unluturk, Ayla Harmanci, Cetin Kocaefe, Bulent O. Yildiz

**Affiliations:** ^1^Department of Internal Medicine, Faculty of Medicine, Hacettepe University, Hacettepe, 06100 Ankara, Turkey; ^2^Endocrinology and Metabolism Unit, Faculty of Medicine, Hacettepe University, Hacettepe, 06100 Ankara, Turkey; ^3^Department of Medical Biology, Faculty of Medicine, Hacettepe University, Hacettepe, 06100 Ankara, Turkey

## Abstract

Polycystic ovary syndrome (PCOS) is the most common endocrine disorder of the women of reproductive age. Familial clustering of PCOS has been consistently reported suggesting that genetic factors play a role in the development of the syndrome although PCOS cases do not exhibit a clear pattern of Mendelian inheritance. It is now well established that PCOS represents a complex trait similar to type-2 diabetes and obesity, and that both inherited and environmental factors contribute to the PCOS pathogenesis. A large number of functional candidate genes have been tested for association or linkage with PCOS phenotypes with more negative than positive findings. Lack of universally accepted diagnostic criteria, difficulties in the assignment of male phenotype, obscurity in the mode of inheritance, and particularly small sample size of the study populations appear to be major limitations for the genetic studies of PCOS. In the near future, utilizing the genome-wide scan approach and the HapMap project will provide a stronger potential for the genetic analysis of the syndrome.

## 1. INTRODUCTION

PCOS is a highly prevalent endocrine disorder affecting approximately 7% of reproductive-aged women [1]. There is no consensus on the diagnostic criteria and definition of PCOS. The most widely used 1990 National Institute of Child Health and Human Development (NICHD) conference diagnostic criteria include (i) clinical and/or biochemical signs of hyperandrogenism, (ii) oligo-ovulation, and (iii) exclusion of other known disorders such as Cushing's syndrome, hyperprolactinemia and nonclassic adrenal hyperplasia [2]. A recent expert meeting sponsored by European Society of Human Reproduction and Embryology (ESHRE)/American Society for Reproductive Medicine (ASRM) suggested that the definition of PCOS should include two of the following three criteria: (i) oligo- and/or anovulation, (ii) clinical and/or biochemical signs of hyperandrogenism, (iii) polycystic ovaries on ultrasonography, and exclusion of related disorders [3, 4].

Patients with PCOS have several interrelated characteristics including hyperandrogenism, altered gonadotropin dynamics, chronic anovulation, polycystic ovaries, and insulin resistance. The syndrome has a significant reproductive and metabolic impact, and is associated with increased risk of type-2 diabetes, dyslipidemia, cardiovascular disease (CVD), and endometrial carcinoma [5–7]. Overall, PCOS can be viewed as a heterogeneous androgen excess disorder with varying degrees of gonadotropic and metabolic abnormalities. Development of PCOS may require the interaction of multiple inherited and environmental factors. Herein, we will briefly overview the available evidence regarding the genetic basis of PCOS.

## 2. FAMILIAL AGGREGATION OF THE SYNDROME

The familial aggregation of PCOS phenotypes and of associated metabolic and reproductive abnormalities has been long noted [8]. While clustering of cases in families strongly support the role of genetic factors in the development of PCOS, heterogeneity of phenotypic features in different families and even within the same family underscores the importance of the environmental contribution. PCOS appears to be a common and complex trait and the exact pattern of inheritance is yet to be fully explained [22, 23]. Family studies of PCOS investigated mainly ovarian morphology, menstrual irregularities, symptoms of hyperandrogenism and hyperandrogenemia [11–14, 17, 20, 22, 24]. The results of these studies are summarized in Table 1.

Cooper et al., in their large study, first described that the incidence of oligomenorrhea and polycystic ovaries is increased in first-degree relatives of PCOS patients compared with the controls [8]. Although male relatives were not specifically studied, a questionnaire revealed that they were noted to have increased pilosity. Additionally, in this study the proposed mechanism of inheritance was autosomal dominant with decreased penetrance [8].

Givens et al. have reported a series of family-based studies, using as diagnostic criteria consisting of hirsutism, oligomenorrhea, and enlarged ovaries [9, 10]. They found familial aggregation of hyperandrogenic and metabolic disorders. These studies were the first to reveal some of the severe metabolic sequelae, such as diabetes mellitus, insulin resistance, lipid abnormalities, hypertension, and arteriosclerosis. They also underscored the variability of phenotype in PCOS, even within the same kindred. Oligospermia and increased LH secretion were found in some of the male subjects of the study participants, suggesting an X-linked pattern of inheritance [9, 10].

Hague et al. utilized high-resolution ultrasonography to identify polycystic ovaries in 61 women with menstrual disturbances, hyperandrogenism, obesity, and infertility as well as in their first-degree female relatives [13]. They found that 67% of the mothers and 87% of the sisters of probands were affected [13]. In this study no attempt was made to identify a male phenotype.

Ferriman and Purdie studied a large group of hirsute women with and without oligomenorrhea and their families [11]. They reported a higher prevalence of hirsutism, oligomenorrhea and infertility in first-degree relatives compared with nonhirsute control women. A questionnaire revealed an increased baldness in male relatives [11]. Affected female and male family members were not systematically characterized in this study.

Lunde et al. studied families of 132 Norwegian women identified on the basis of an ovarian wedge resection, who also had two or more of the following symptoms: menstrual irregularity, hirsutism, infertility, and/or obesity [12]. They also compared these women with controls and their families, and found a significantly higher percentage of PCOS-related symptoms in the first-degree female relatives of PCOS patients and observed a significantly higher percentage of premature balding and increased pilosity among male relatives [12].

Using NICHD criteria for the diagnosis of PCOS, Kahsar-Miller et al. reported the rates of PCOS in mothers and sisters of patients with PCOS as 24% and 32%, respectively [20]. Legro et al. showed that 22% of reproductive aged sisters of women with PCOS fulfilled the diagnostic criteria of PCOS, whereas 24% had increased *T* and DHEAS values with regular menstrual cycles [17]. We have reported 16% and 8% PCOS prevalence rates in sisters and mothers of Turkish PCOS patients, respectively [21].

Recently, quantitative phenotypes related to hyperandrogenemia and glucose homeostasis are also shown to be heritable in PCOS. Evidence for heritability of metabolic phenotypes such as beta cell function and insulin resistance was reported in family studies of PCOS. Studying the families of five patients with PCOS, Norman et al. reported that increased insulin levels were common among first-degree relatives [15]. Colilla et al. noted that there was a heritable component of beta cell dysfunction in families of women with PCOS [16]. Legro et al. reported that affected sisters of women with PCOS (who fulfill criteria for the diagnosis of PCOS, and those with hyperandrogenemia) had high insulin levels and low fasting glucose to insulin ratios [18], and the brothers of women with PCOS had increased dehydroepiandrosterone (DHEAS) levels [19]. We have reported that mothers and fathers of PCOS patients have increased prevalence of glucose intolerance and type-2 diabetes whereas brothers and sisters show insulin resistance compared to age- and BMI-matched healthy controls [21].

## 3. METHODS USED IN GENETIC STUDIES OF PCOS

Two mainstream approaches employed to identify a genetic locus for PCOS are (i) association studies where a predisposing allele is expected to be encountered more frequently in the effected population than the normal individuals and (ii) linkage studies where the probands and their families are investigated to determine if particular genomic landmarks are distributed independently or in linkage (together) with the phenotype. While the mode of inheritance is not required for the association studies, it requires that a relatively large set of individuals are needed for a clear conclusion. The canonical linkage studies require that the mode of inheritance should be known for the analysis procedure. These studies are quite robust to identify single genes causing Mendelian disorders but are poorly suited to the genetic architecture of complex traits such as PCOS. The variable penetrance and expressivity are two main factors that are complicating both association and linkage methods (reviewed in [25]).

The frequency of the genomic landmarks used in linkage studies defines the resolution and the mapping power of the study. The whole genome scan approach utilizing the SNP (single nucleotide polymorphism) microarray gene-chip technology brings the highest resolution in genetic mapping [26]. Yet, there is no PCOS study published with this technique. Furthermore the HapMap project brings further power to association studies by genotyping over a million SNPs and characterizing genetic variation patterns in linkage disequilibrium [27].

## 4. CANDIDATE GENES IN PCOS

### 4.1. Genes involved in ovarian and adrenal steroidogenesis

**Figure 1:**
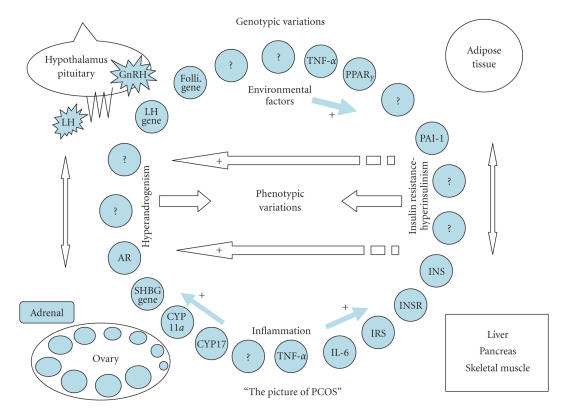
PCOS is a complex genetic syndrome. A dysregulation of androgen synthesis plays a key role in the pathogenesis of PCOS. This dysregulation may be triggered by genomic variants related to hyperandrogenism and environmental factors, such as sedentary life-style and dietary habits. The hyperandrogenemic condition causes follicles not to grow as much as dominant follicle and leads oligo/anovulation. The progesterone peak does not occur through luteal phase of menstrual cycle, and the frequency and amplitude of GnRH pulses are increased, which in turn cause the secretion of LH to increase. By means of increasing LH levels, androgen synthesis and secretion are stimulated in the ovaries and adrenals. On the other hand, the inherited insulin resistance leads a hyperinsulinemic condition causing androgen synthesis to increase and SHBG synthesis to decrease. Additionally, obesity that is inherited and/or acquired could cause a chronic inflammation via secreting inflammatory cytokines from adipose tissue, which stimulates androgen sythesis and increases insulin resistance. As a consequence, interactions among ovary, hypothalamus-pituitary, adrenal, adipose tissue, liver, skeletal muscle and $\tilde{\beta }$-cells of pancreas draw the picture of PCOS. Environmental factors and genetic variations constitute the *phenotypic variability*/colors of the picture.

The most common biochemical abnormality in women with PCOS is hyperandrogenemia (Figure 1). For this reason, researchers have long been trying to find a linkage or an association between PCOS and the genes involved in the androgen biosynthetic pathway. The most relevant genes involved in steroidogenesis, CYP11a, CYP21, CYP17, and CYP19, along with their controversial properties are discussed below.

#### 4.1.1. CYP11a

Adrenal and ovarian steroidogenesis start with the conversion of cholesterol into progesterone, which is catalyzed by the P450 cytochrome side chain cleavage enzyme encoded by CYP11a located at 15q24 [28]. This conversion is a rate limiting step of steroidogenesis. Gharani et al. conducted an association study of 97 women with PCOS and showed a significant association between serum testosterone levels and the alleles of the CYP11a with a 5′ untranslated region (UTR) consisting of repeats of a (tttta)_*n*_ pentanucleotide, a VNTR (variable number tandem repeat) polymorphism [29]. Two independent case-control studies from Greece [30] and China [31], respectively, confirmed these findings in support of the encouraging evidence for the association between CYP11a and PCOS.

Although these studies proposed that allelic variants of CYP11a have a role in the etiology of hyperandrogenemia and/or PCOS, subsequent studies including one with a very large sample size from United Kingdom and Finland [32] have failed to find a significant linkage or association between this gene locus and/or its VNTR alleles and PCOS [32–36].

Above all, CYP11a, with its associated polymorphisms, remains, at least in part, a potential candidate gene for the pathogenesis of PCOS, and further investigations are required due to these controversial results.

#### 4.1.2. CYP21

One of the main steps in adrenal and ovarian steroidogenesis is the conversion of 17-hydroxyprogesterone into 11-deoxycortisol, which is catalyzed by the 21-hydroxylase enzyme encoded by CYP21. The deficiency of this enzyme, which is inherited by an autosomal recessive trait, is responsible for most cases of congenital adrenal hyperplasia, and increased serum 17-hydroxyprogesterone levels are correlated with its deficiency. Women with functional hyperandrogenism or PCOS have an increased serum 17-hydroxyprogesterone response to ACTH stimulation as a common finding [37, 38]. Furthermore, patients having both heterozygote CYP21 mutations and clinical symptoms exhibit a PCOS-like phenotype [39]. Accordingly, mutations of CYP21 have been investigated as a candidate gene in patients with PCOS. Witchel et al. showed that children with premature pubarche and adolescent girls with hyperandrogenism were heterozygous for mutations in CYP21 [39, 40]. However, Escobar-Morreale et al. found no clear concordance between the CYP21 genotype and the functional origin of androgen excess [41] and Glintborg et al. could not find such a concordance in women with idiopathic hirsutism and PCOS [42]. More recently, Witchel et al. conducted a case-control study of 114 PCOS patients, which indicated no significant differences in the allele frequency of CYP21 mutations between PCOS patients with and without androgen excess and controls [43]. Overall, CYP21 and associated mutations seem not to play a key role in the development of PCOS.

#### 4.1.3. CYP17

Other rate limiting steps of adrenal and ovarian androgen biosynthesis are the conversion of pregnenolone and progesterone into 17-hydroxypregnenolone and 17-hydroxyprogesterone, respectively, and of these steroids into dehydro-epiandrosterone and androstenedione, which is catalyzed by the P450c17*α* enzyme. This enzyme has both 17*α*-hydroxylase and 17,20-lyase activities and is encoded by CYP17 located at 10q24.3 [44, 45]. It was initially proposed that an exaggerated adrenal and ovarian responsiveness and increased P450c17*α* enzyme activity were responsible for enhanced androgen levels in patients with PCOS and functional hyperandrogenism [46, 47]. Escobar-Morreale et al. also suggested that most of the hyperandrogenic women have increased P450c17*α* activity in adrenal and ovarian sites [37, 48]. In accordance with these studies, Wickenheisser et al. reported increased P450c17*α* expression and enzymatic activity in ovarian theca cells from women with PCOS as well as increased transactivation of the CYP17 promoter [49, 50]. Moreover, they reported for the first time that CYP17 expression is dysregulated at the level of mRNA stability in PCOS theca cells [51].

In another study, Carey et al. identified a rare T/C single nucleotide polymorphism (SNP) in the promoter region of CYP17 increasing the susceptibility to develop PCOS [52]. Even though Diamanti-Kandarakis et al. confirmed this finding in Greek patients with PCOS [53], subsequent more comprehensive studies have failed to detect a significant linkage or association between CYP17 and PCOS [35, 54–58].

Although the available evidence suggests that CYP17 is not a strong candidate gene for PCOS, we should also note that posttranslational regulation of this gene product might play a role in the pathophysiology of PCOS. Because serine phosphorylation is involved in the posttranslational regulation of 17,20-lyase activity [59], it was proposed that posttranscriptional hyperphosphorylation of the serine residues of P450c17*α* by a defective serine kinase might increase the 17,20-lyase activity of this enzyme [59, 60]. Interestingly, it was demonstrated that serine phosphorylation of the *β*-chain of the insulin receptor causes insulin resistance in vitro [61, 62]. Another interesting finding is that 50% of PCOS women who had both hyperandrogenism and insulin resistance had hyperphosphorylated serine residues on their insulin receptors [63]. Therefore, that a single defect produces both the insulin resistance and the hyperandrogenism in some of the PCOS women was postulated [64]. Altogether, these data provide an encouraging evidence to support the serine kinase hypothesis of PCOS; however, this hypothesis is still to be proved.

#### 4.1.4. CYP19

An enzyme complex, called aromatase, converts C19 steroids (androgens) to C18 steroids (estrogens). This enzyme complex is composed of the cytochrome P450 aromatase (P450arom) and the NADPH cytochrome P450 reductase [65], and P450arom is encoded by CYP19 located at 15p21.1 [66, 67]. Aromatase deficiency has been reported in a number of hyperandrogenic patients [68, 69]. Immunohisto-chemical studies of polycystic ovaries could not reveal any aromatase activity in antral follicles of various sizes [70]. Erickson et al. demonstrated that granulosa cells obtained from medium-sized follicles of women with PCOS have little aromatase activity [71]. Similarly, Jakimiuk et al. showed that when compared to the control follicles, all PCOS follicles contained low levels of P450arom mRNA, estradiol, and lower aromatase stimulating bioactivity [72]. These findings indicate that the aromatase activity might be decreased in follicles from patients with PCOS, and that the possible androgen excess resulting from this decreased activity might contribute to abnormal follicle development. Whether the CYP19 is a candidate gene for the pathogenesis of PCOS or of hyperandrogenism has, therefore, been investigated. Linkage and mutation screening studies did not reveal any evidence that variation at the CYP19 locus participates in the etiology of PCOS [29, 35, 73]. However, association studies utilizing SNPs and haplotypes showed association with PCOS symptom score and serum testosterone levels [74, 75].

### 4.2. Genes involved in steroid hormone effects

#### 4.2.1. Androgen receptor gene

All androgens operate through the androgen receptor and this receptor belongs to a family of nuclear transcription factors. The androgen receptor is encoded by a gene (AR) located at Xq11-12 [76] and is composed of three functional domains: the transactivation domain, the DNA binding domain, and the ligand-binding domain. A VNTR polymorphism consisting of CAG repeats (from 11 to 38 repeats, an average of 20) in exon-1, encoding a polyglutamine chain in the *N*-terminal transactivation domain, is embedded in AR [77]. The transcriptional activity of androgen receptor is inversely correlated with the number of CAG repeats [78]. Variations of these repeats, even within the normal polymorphic range (11–38 CAGs), have been related to various disorders associated with low- or high-androgenic activities [79–82]. Therefore, decreased number of CAG repeats with an increased androgen receptor activity could explain some of the PCOS phenotype exhibiting the normal serum androgen levels and hyperandrogenism symptoms [83]. Nevertheless, Urbanek et al. could not find any association between this VNTR and PCOS [35]. Similarly, Mifsud et al. found no differences between the anovulatory PCOS patients and controls for the distribution of the CAG repeats [83]. On the contrary, Hickey et al. demonstrated a significantly greater frequency of alleles with longer CAG repeats (> 22 repeats) for infertile PCOS patients compared with fertile women [84]. Indeed, both of these studies indicated an association between the testosterone levels and CAG repeats in AR, albeit with different results. Confirming the reports of Urbanek et al. [35] and Mifsud et al. [83], in a more recent study conducted in Finland, Jääskeläinen et al. reported that CAG repeats of AR are not the major determinants of PCOS [85]. They also found no correlation between this VNTR and body mass index or serum testosterone levels [85]. As a result, no convincing evidence exists about the role of AR in the pathogenesis of PCOS.

#### 4.2.2. Sex hormone-binding globulin gene

SHBG regulates the access of androgens to target tissues. Serum SHBG levels are commonly low in patients with hyperandrogenism, especially in association with PCOS, which contributes to increased tissue androgen availability [86]. Human SHBG is composed of a homodimeric glycoprotein produced by hepatocytes and is encoded by a 4-kb gene at the 17p12-p13 [87, 88]. A pentanucleotide repeat polymorphism, (TAAAA)_*n*_, at the promoter of human *SHBG gene* has been described [89]. This polymorphism has also been demonstrated to influence the transcriptional activity of SHBG gene [89]. Under the light of these data, it has been proposed that this functional polymorphism could contribute to individual diversities in plasma SHBG levels and thus influence the access of androgens to target tissues [90]. For this reason, Xita et al. investigated whether the (TAAAA)_*n*_ polymorphism of the *SHBG gene* is associated with PCOS and whether polymorphic variants of the gene are related to serum SHBG levels in women with PCOS. They reported a significant association between this repeat polymorphism and PCOS in a Greek population [90]. In this study, PCOS patients were frequently carrying more than 8 repeats while nonhyperandrogenic controls presented with a higher frequency of alleles carried fewer than 8 repeats. Besides, PCOS patients carrying the longer allele genotypes had lower SHBG levels [90]. In accordance with the latter result, Cousin et al. recently demonstrated in hirsute women that longer (TAAAA)_*n*_ alleles caused serum SHBG levels to be decreased when compared with six repeat alleles [91]. They also identified that an SNP (Asp327Asn) in exon 8 of the *SHBG gene* increases the SHBG half-life as well as having a strong disequilibrium linkage with the eight (TAAAA)_*n*_ polymorphism [91]. Although Urbanek et al. could not find any association or linkage between a marker close to the SHBG locus and PCOS [35], based on the available evidence it could be concluded that *SHBG gene* is a potential candidate gene in the pathogenesis of PCOS.

### 4.3. Genes involved in gonadotropin action and regulation

#### 4.3.1. LH and its receptor genes

Both increased LH levels and altered LH action are frequently observed in PCOS patients, and these abnormalities are associated with anovulation through, at least in part, an adverse effect of LH on oocyte maturation [92, 93]. Therefore, the gene encoding the *β*-subunit of LH, responsible for LH specificity, has been explored in PCOS patients.

Initially, an immunologically abnormal form of LH with two-point mutations, Trp8Arg and Ilg15Thr, in the *LH β-subunit gene* was identified [94]. Afterwards, it was demonstrated that these mutations are universally common polymorphisms with 15% prevalence worldwide [95]. In addition, these mutations produced structural changes in the variant LH molecules (v-LH) [96] and caused v-LH to have an increased in vitro activity and a decreased in vivo half life compared to that of nonmutant form [97]; however, in vivo activity of v-LH could not be explained. In order to understand the relevance of v-LH in patients with PCOS, Rajkhowa et al. explored the implication of v-LH in both healthy women and PCOS patients and found that the occurrence of these mutations in *LH β-subunit gene* was not higher in PCOS compared with healthy women [98]. On the other hand, subgroup analysis of this study revealed that obese PCOS patients had a higher frequency of the heterozygous v-LH compared with the obese controls [98]. Contrary to the latter finding, Tapanainen et al. in their multicenter study reported that the obese PCOS patients from the Netherlands, Finland, and the United States, but not from the United Kingdom have a lower frequency of this variant form [99]. Although this finding suggests that v-LH somehow protects obese women from developing PCOS, other studies failed to find any association with PCOS [95, 100, 101].

Another identified variant form of LH was due to a single missense mutation, Gly102Ser, in the *LH β-subunit gene* [102]. Ramanujam et al. studied this mutation in 176 patients with menstrual disorders and 200 normal ovulatory Singapore Chinese women and found it present in only seven patients with menstrual disorders [100]. Recently, Takahashi et al. demonstrated that numerous SNPs in the promoter region of the *LH β-subunit gene* were more frequent in patients with ovulatory disorders including PCOS than normal ovulatory women [103].

The hypothesis that an activating mutation in the *LH receptor gene* might be a cause of hyperandrogenism in patients with PCOS having normal serum LH concentrations and raised androgen levels was tested using linkage analysis in families with multiple cases of PCOS, and five families in whom polymorphic markers close to the *LH receptor gene* segregate with the syndrome were identified [104]. Nevertheless, in a subsequent preliminary study, these authors did not find any mutations after gene sequencing in these affected families [104]. Likewise, Urbanek et al. reported negative results in their study in which total of 37 potential candidate genes were examined in 150 families with PCOS [35].

In summary, the functional effects of these v-LHs remain unclear and seem not to play a key role in PCOS pathogenesis or female infertility, but further studies are required to determine the physiological and pathophysiological significance of this LH variant.

#### 4.3.2. Follistatin gene

Follistatin, a monomeric glycoprotein encoded by a single gene, is structurally unrelated to the TGF-*β* superfamily, but is linked functionally through its role as a high-affinity binding protein for activin [105]. Activin, a dimeric glycoprotein belonging to the TGF-*β* superfamily, induces FSH and insulin secretion, ovarian follicular maturation and inhibits LH-stimulated ovarian androgen production [105]. As an activin-binding protein, follistatin can reverse each of these activin-induced responses in vitro and in vivo [105–108]. Actually, overexpression of follistatin in transgenic mice resulted in suppression of serum levels of FSH and arrested ovarian folliculogenesis [106]. Excessive activin neutralization due to increased follistatin may, therefore, reduce FSH concentrations, arrest follicular maturation, increase androgen production, and impair insulin release. Because all of these changes are typical features of PCOS [109], *follistatin gene* has been explored as a candidate gene in PCOS.

Initially, Urbanek et al. examined a total of 37 potential candidate genes in 150 families with PCOS, as mentioned above, and reported statistically significant linkage only between the *follistatin gene* and PCOS [35]. However, subsequent more comprehensive *follistatin gene* studies conducted by the same authors have not obtained any significant linkage [110]. In order to detect variation in the *follistatin gene*, they made sequence analysis of this gene in 85 women of 19 families of PCOS patients and identified sequence variants at 17 sites. Nevertheless, only one polymorphism of these sites was common, and in addition, the site of this polymorphism was not translated [110]. They also reported similar expression of the *follistatin gene* mRNA in cultured fibroblasts from PCOS and control women [110]. Likewise, two studies including patients with PCOS from Singapore [111] and Spain [112] could not find any significant mutations in coding regions of the *follistatin gene*.

### 4.4. Genes involved in insulin action and secretion

Almost two decades ago, it was demonstrated that most women with PCOS either obese or nonobese, compared with normal women, exhibited variable degrees of insulin resistance and compensatory hyperinsulinemia [113]. Subsequently convincing evidence has been started to accumulate, and at present, it is well known that hyperinsulinemia and insulin resistance are common features of PCOS patients [64] (Figure 1). Therefore, numerous genes involved in insulin action and secretion have been explored as candidate genes in PCOS pathogenesis. The *insulin gene (INS)*, the *insulin receptor gene (INSR)*, the *insulin receptor substrate genes (IRSs)* and *calpain-10 gene (CAPN10)*, the most relevant genes involved in insulin action and secretion, are discussed below with their controversial results.

#### 4.4.1. The insulin gene

The *INS* is located between the genes for tyrosine hydroxylase and for IGF-II at 11p15.5, and includes variable tandem repeats (VNTR) embedded at the 5′ regulatory region of INS [114]. The VNTR polymorphism regulates the transcriptional rate of the *INS* [115] and probably that of the gene encoding IGF-II [116]. The number of the repeats of the *INS* VNTR ranges from 26 to 200, and due to this feature *INS* VNTR polymorphism has three size classes. Class-I alleles compose the shorter polymorphic region, consisting of an average length of 40 repeats. Class-II alleles have an average length of 80 repeat units and are uncommon in Caucasian. Class-III alleles compose the longest polymorphic region having an average of 157 repeats [117]. Transcriptional activity of the longer polymorphic region is greater than that of the shorter one [115]. Besides their effect on regulating *INS* expression, they have been implicated in the pathogenesis of type-2 diabetes mellitus in many studies [118, 119].

It is not known whether the hyperinsulinemia detected in PCOS is an outcome of primary insulin resistance or the direct effect of pancreatic *β*-cell disorder, whereas defects in both insulin action [113, 120] and in pancreatic *β*-cell function [121, 122] have been reported. Therefore, in order to clarify the genetic basis of PCOS and to determine its association with defects in insulin secretion and action, firstly, Waterworth et al. evaluated the linkage and association of the *INS* VNTR polymorphisms in families with affected members with PCOS or male-pattern baldness [123]. They have found an association between PCOS and allelic variation at the *INS* VNTR locus in three separate populations [123]. Furthermore, they found that class III alleles, especially the III/III genotypes, were associated with anovulatory PCOS in two independent populations and were more frequent among women with polycystic ovaries with symptoms than those without symptoms [123]. In addition, in the same study, it was shown that the geometric mean of fasting serum insulin concentrations was significantly higher in families with evidence of linkage than in families with no evidence of linkage [123]. These data support the idea that the VNTR polymorphisms have a functional role on the establishment of hyperinsulinemia and/or insulin resistance component phenotypes in PCOS. The same group also reported that class III alleles were transmitted significantly more common from fathers than from mothers to affected daughter, suggesting a “parent of origin” effect in the transmission of alleles [123]. The latter finding was confirmed by Eaves et al. [124]. In support of this evidence, Michelmore et al. demonstrated that class III alleles, III/III genotype, and paternal class III allele transmissions were significantly related to increased number of PCOS features and to reduced insulin sensitivity among women with PCOS [125].

In a more comprehensive study, however, Urbanek et al. could not find any evidence for the linkage of *INS* and PCOS and for the association of the class III allele and of hyperandrogenemia [35]. But the difference of this study from other previous studies where the ultrasonographic findings were more commonly used was that the NIHCD selection criteria were used. Likewise, Calvo et al. studied the *INS* VNTR polymorphisms in Spanish women with hyperandrogenemia and failed to show any association compared to the controls [126]. Similarly, Vanková et al. studied the association of *INS* VNTR polymorphisms with PCOS in Czech women and failed to find any association relevant to VNTR and PCOS [127]. Using different selection criteria, studying patients with variable ethnic and geographical backgrounds, selection bias, and most importantly working with small to, at best, modest sample sizes might explain the presence of these conflicting results. Consistently, studying associations with a small sample size is known to be a major risk factor for the generation of results that cannot be replicated on consecutive examinations [128]. As a solution to this problem, more recently, Powell et al. rebutted the relationship between the *INS* VNTR and PCOS, using several complementary analytical approaches, including case-control, family-based, and quantitative trait association methods in more than 3500 subjects from United Kingdom and Finland [129].

#### 4.4.2. INSR

The insulin receptor is a heterotetrameric glycoprotein composed of two *α* and two *β*-subunits and is encoded by the *INSR* located at the chromosome 19 [130]. Several studies were conducted to identify whether the mutations of *INSR* could explain insulin resistance in women with PCOS. Initially, direct sequencing of *INSR* from two obese women with PCOS did not reveal any mutations [131]. Consequently, Conway et al. [132] analyzed the sequence of the tyrosine kinase domain of *INSR* in 22 hyperinsulinemic patients with PCOS and Talbot et al. [133] investigated the mutations by molecular scanning of the entire coding region of INS in 24 hyperinsulinemic women with PCOS, and none of these groups detected any significant mutations related to insulin resistance in PCOS [133]. In addition, Urbanek et al., using the dinucleotide repeat marker, D19S884, found evidence for the association of the *INSR* with PCOS in their TDT analysis, but after correction for multiple testing, this finding lost its statistical significance [35]. However, using the same marker, Tucci et al. proved the association with *INSR* in women with PCOS [134]. Nevertheless, this association could not be confirmed in a consecutive study from the Mediterranean area [135]. More recently, a comprehensive study published by Urbanek et al. demonstrated a linkage with PCOS in a group of well-characterized 367 families including individuals predominantly of European origin with PCOS [136]. A broad region of the chromosome 19p13.2 was investigated and the strongest evidence for association was found with D19S884, supporting the previous findings by these authors [136].

In another study, Siegel et al. examined an SNP at the tyrosine kinase domain of *INSR* and found an association in lean patients with PCOS. This SNP could be a susceptibility variant for PCOS, or it can be a result of linkage disequilibrium with another *INSR* polymorphism, but the association is pending confirmation [137].

#### 4.4.3. Insulin receptor substrate proteins

Activation of the insulin receptor following insulin binding requires the autophosphorylation of the *β*-subunit of the insulin receptor [138]. The consequent tyrosine kinase activity generated after autophosphorylation phosphorylates insulin receptor substrates (IRS), such as IRS-1 and IRS-2 [64]. Afterwards, IRS-1 and IRS-2 bind and activate downstream effectors, such as phosphoinositide 3-kinase, to promote the metabolic and mitogenic actions of insulin. When IRS-1 is dysfunctional, IRS-2 is the main messenger for the intracellular transmission of the insulin signal, but it requires a higher insulin concentration for activation [139].

Several polymorphisms of IRS1 and IRS2 genes (*IRS1* and *IRS2*) have been implicated in insulin resistance. The Gly972Arg polymorphism for *IRS-1* and Gly1057Asp for *IRS-2* have been shown to increase susceptibility to type-2 diabetes mellitus [140, 141]. Although initially no evidence for linkage or association with PCOS was found with *IRS-1* in a family-based study conducted by Urbanek et al. [35], the potential roles of these SNPs of IRS genes in insulin resistance have further been investigated in PCOS. Sir-Petermann et al. reported a higher frequency of the Arg972 *IRS-1* allele in PCOS patients in Chilean population [142]. Contrary to this report, El Mkadem et al. could not find any differences in the distribution of *IRS-1* Gly972Arg and *IRS-2* Gly1057Asp alleles in PCOS patients and controls; however, they demonstrated that the Gly972Arg *IRS-1* was more prevalent in insulin-resistant patients compared with the noninsulin resistant patients or control subjects [143]. Besides, they showed gene-dosage effects on fasting insulin levels for Gly972Arg *IRS-1* and on 2-h plasma glucose levels during oral glucose tolerance test (OGTT) for Gly1057Asp *IRS-2* in women with PCOS, while no novel mutations have been found in these genes by direct sequencing [143]. Ehrmann et al. investigated the influences of Gly972Arg *IRS-1* and of Gly1057Asp *IRS-2* polymorphisms in nondiabetic women with PCOS [144]. Although the *IRS-1* genotype was not found to be associated with any clinical or hormonal measures in nondiabetic PCOS subjects, carrying *IRS-2* Gly/Gly genotype was associated with significantly higher glucose levels in 2-h OGTT compared with carrying Gly/Asp and Asp/Asp genotypes [144]. In this study, the association of Gly/Asp genotype with lower glucose levels during 2-h OGTT [144] was just contrary to the report of El Mkadem et al. [143]. Confirming the report of El Mkadem et al. [143], Villuendas et al. showed that these polymorphisms had an equal distribution pattern among PCOS patients and controls from Spain [145]. These investigators also found that carrying the Arg972 allele for *IRS-1* and carrying the homozygous Gly1057 for *IRS-2* present negative effects on glucose homeostasis compared with carrying the homozygous Gly972 alleles for *IRS-1* and one or two Asp1057 alleles for *IRS-2*, respectively [145].

In a recent study, Dilek et al. reported a higher frequency of the Gly972Arg polymorphism for *IRS-1* in Turkish women with PCOS [146] in accordance with the data of Sir-Petermann et al. [142]. Moreover, similar to the data obtained by El Mkadem et al. [143] and Villuendas et al. [145], they found that the Gly972Arg carriers were more obese, more insulin-resistant and had higher fasting insulin levels when compared with the other PCOS patients and controls [146]. These investigators also studied the same Turkish PCOS patients for the potential differential effects of metformin therapy on the basis of *IRS-1* genotype [147]. In this study, metformin lowered the LH, DHEAS, total testosterone, and fasting insulin levels and decreased insulin resistance and free testosterone index in Gly972Arg-negative PCOS women more effectively and significantly when compared with the Gly972Arg-positive women [147]. These findings could be taken as an indirect indicator of the relationship between the *IRS-1* genotype and the insulin resistance phenotype of PCOS. Interestingly, some of the proposed mechanisms for the action of metformin at cellular level are that it might augment the tyrosine phosphorylation of the insulin receptor *β*-subunit and IRS proteins and also increase the insulin-dependent and nondependent cellular glucose uptake through the family of glucose transporter proteins [148]. In accordance with these proposals, Ertunc et al. hypothesized that variant IRS-1 proteins might not be able to transmit signals and thus might not be able to increase the glucose uptake into the muscle and adipose cells [147]. This hypothesis may, at least in part, be an explanation of the association of *IRS-1* genotype with insulin resistance in some of PCOS patients; however these data are yet to be confirmed.

Overall, the association studies of *IRS*s with PCOS and insulin resistance phenotype of this syndrome exhibit many conflicting properties as seen with the other candidate genes for PCOS. We should emphasize however that these *IRS* polymorphisms seem to be associated separately with insulin resistance rather than PCOS in these studies. Therefore, more comprehensive studies are required to solve the puzzle of relations among these genes and PCOS.

#### 4.4.4. Calpain10 gene

Calpain-10 is a cysteine protease that plays a role in insulin secretion and action [149], and genetic studies have shown that variation in the gene (*CAPN10*) encoding calpain-10 is associated with type-2 diabetes [150]. Due to the fact that PCOS and type-2 diabetes share a number of etiologic factors [64, 151], Ehrmann et al. sought to determine whether variation in the *CAPN10* is associated with quantitative traits related to the pathogenesis of PCOS and type-2 diabetes [152]. They found an association between the 112/121 haplotype of this gene and higher insulin levels in African-American women and an increased risk of PCOS in both African-American and white women [152]. Gonzales et al. investigated whether four SNPs (SNP-19, SNP-43, SNP-44, and SNP-63) of the *CAPN10* were associated with PCOS [153, 154]. In support of the latter, they reported in their consecutive studies that SNP-44 of the gene is associated with PCOS in Spanish women [153, 154]. Nevertheless, using the same four SNPs, Haddad et al. could not confirm any association with PCOS in a more comprehensive study [155]. Likewise, Escobar-Morreale et al. studied three SNPs (SNP-43, SNP-44, and SNP-45) of *CAPN10* and reported no association between any of those and PCOS [156]. These conflicting results suggest that definitive conclusions could only be drawn by studying larger populations probably including thousands rather than hundreds of subjects.

### 4.5. Genes involved in energy homeostasis and the relevance of peroxisome proliferator-activated receptor-*γ* gene

#### 4.5.1. The genes of leptin, leptin receptor, and adiponectin

Although the adipose tissue has long been regarded as an inert and passive type of connective tissue that stores and releases energy, during the last decade it has been recognized that the adipose tissue is not only a connective tissue but is also one of the active endocrine organs which secretes a wide variety of products called as adipocytokines [157]. Due to the fact that a substantial proportion of women with PCOS are overweight, many are obese and some are extremely obese, the genes of the most popular adipocytokines such as leptin and adiponectin have been investigated as candidate genes in the pathogenesis of PCOS.

Oksanen et al. sequenced the *leptin gene* in a small group of PCOS patients, but failed to detect any mutations of the coding exons [158]. In this study, the investigators also sequenced the leptin receptor gene and found previously identified amino acid variants in exons 2, 4, and 12 as well as the pentanucleotide insertion in the 3′-untranslated region [158]. However, the allele frequencies of these polymorphisms did not differ from those in the general population [158].

After the sequence polymorphisms of *adiponectin gene* identified in humans, most studies have focused on two polymorphisms, T45G in exon 2 and G276T in intron 2. It was demonstrated consistently that these polymorphisms associate with obesity, insulin resistance, and the risk of type-2 diabetes [159–162]. In a study assessing the association of PCOS with 15 genomic variants previously described to influence insulin resistance, obesity, and/or type-2 diabetes mellitus, San Millán et al. failed to find any association between PCOS and these two common polymorphisms of the *adiponectin gene* [163]. Concurrently, Panidis et al. investigated the possible association of the T45G *adiponectin gene* polymorphisms with PCOS [164]. Although the frequency of GG, TT, and TG genotypes were similar between women with PCOS and controls, when the frequencies of GG and TG genotypes assessed together, a significant difference was observed between the groups [164]. This study reported no significant relation between the T45G adiponectin gene polymorphism and circulating adiponectin levels, obesity, and the metabolic features of PCOS; however, it was observed that the carriers of the G allele had a tendency for lower serum adiponectin levels in PCOS group [164]. More recently, two different studies from Greece [165] and Spain [166] rebutted the probability that the T45G and G276T polymorphisms of *adiponectin gene* could be associated with PCOS. Additionally, these studies reported the conflicting results about effects of these SNPs on adiponectin and hormonal variables [165, 166].

Taken together these data suggest that, different from the other insulin resistant disorders; the *adiponectin gene* seems not to play a causative role in the pathogenesis of PCOS. However, the polymorphisms of the *adiponectin gene* may, at least in part, have a role in the phenotypic variability of PCOS.

#### 4.5.2. PPAR-*γ* gene

PPAR-*γ* is a transcription factor involved in adipogenesis, energy metabolism and a functional receptor for thiazolidinediones (TZDs) introduced as insulin-sensitizing agents [167]. Additionally, PPAR-*γ* gene *(PPAR-γ)* located on 3p25 [168] is a candidate gene for the regulation of adipose tissue metabolism in humans and also a susceptibility gene for the development of both obesity and diabetes [169–171]. PPAR-*γ* agonists, namely TZDs, currently offer an effective treatment option for the management of patients with type-2 diabetes. Since PCOS and type-2 diabetes share certain phenotypic features such as obesity and insulin resistance, several studies investigated the potential therapeutic effects of TZDs on the ovulatory dysfunction, hirsutism, hyperandrogenism and insulin-resistance of PCOS. The vast majority of these clinical trials reported positive results [172–174].

Studies of rare mutations and frequent polymorphisms of *PPAR-γ* have facilitated to improve our knowledge on the biological roles of PPAR-*γ* and its implication in diseases. There are two *PPAR-γ gene* polymorphisms that have been thoroughly investigated in various populations (reviewed in [175]). The first is the silent CAC478-CAT substitution which resides in the exon 6 and the second is a proline to alanine missense variant at the codon 12 of the exon 2. Studies assessing the potential impact of *PPAR-γ gene* on PCOS are discussed below and summarized in Table 3.

In the first study, Urbanek et al. found no evidence for any linkage or association with PCOS for a marker close to the *PPAR-γ* gene [35]. In another study, Hara et al. showed that Caucasian PCOS patients with Pro/Ala alleles are more insulin sensitive than those with Pro/Pro [176]. Recently, Hahn et al. also reported that the Pro12Ala polymorphism of *PPAR-γ* are associated with increased insulin sensitivity as well as lower hirsutism scores in PCOS women [177]. Afterwards, in accordance with these data, Tok et al. reported that carriers of this polymorphism were less insulin-resistant than the Pro12Ala-negative controls and patients with PCOS. The difference between the groups for carrying the polymorphism did not reach significance, probably due to very small sample size [178]. In addition, Korhonen et al. from Finland investigated the Pro12Ala polymorphism in patients with PCOS and demonstrated that the frequency of the variant Ala isoform was reduced with a slight significance in the PCOS group [179]. Furthermore, Yilmaz et al. examined the impact of the Pro12Ala polymorphism on insulin resistance in the first-degree relatives of subjects with PCOS. They found that the frequency of the variant Ala isoform was significantly reduced in the first-degree relatives of PCOS subjects compared to the controls and also demonstrated that the first-degree relatives of PCOS subjects with the Pro12Ala polymorphism were less insulin resistant than the first-degree relatives of PCOS subjects with the Pro12Pro polymorphism [180]. The latter group, in a more recent study, examined the relationship between the Pro12Ala polymorphism and the clinical and hormonal characteristics in women with PCOS. When PCOS subjects with the Pro allele and the Ala allele of *PPAR-γ* were compared, they found that having the Ala allele of this nuclear receptor gene was related with reduced androgen levels, a lower Ferriman-Gallwey score, a lower insulin resistance index, a lower body mass index, and a lower waist-to-hip ratio [181]. However, this type of polymorphism was not found to be associated with PCOS in women from Spain [163], Italy [182] and China [183]. Additionally, in two different studies, Orio et al. have investigated the impact of *PPAR-γ* gene polymorphisms in PCOS pathogenesis. First, they studied the exon-6 C142T allele of PPAR-*γ* and showed a higher frequency in Italian women with PCOS compared to the controls [182]. Subsequently, they investigated the putative influence of *PPAR-γ* gene Pro12Ala polymorphism on the adiponectin levels in PCOS patients and in healthy women. However, they failed to demonstrate any effect of Pro12Ala polymorphism on adiponectin levels in neither group [184].

The limited size of the study populations and the low stringency in diagnostic criteria are the limitations of the genetic association studies seeking for the relevance of the PPARg gene with PCOS and thus, should be interpreted cautiously. Likewise, the lack of a clear-cut association to the polymorphisms does not rule out the implications of PPARg on the pathogenesis of PCOS. Even though genetic association studies do not clearly establish any link between PCOS and *PPAR-γ* polymorphisms, functional investigations still point out the suspicious role of *PPAR-γ* on PCOS. Seto-Young et al. demontrated that PPAR-*γ* ligands stimulate the steroidogenesis on human ovarian steroidogenic tissue [191]. Pioglitazone treatment increased growth hormone secretion and stimulated levels in PCOS patients in a randomized trial [192]. Authors comment that this action might be a function of improved insulin sensitivity [192]. While these functional studies implicate the relevance of *PPAR-γ* function to PCOS, the molecular mechanisms to explain the beneficial action of PPAR-*γ* activators on the clinical picture of PCOS are still obscure. This effect may be a consequence of potentiating the insulin sensitivity on the periphery and the gonads [192]. Jansen et al. employed a more mechanistic approach and compared the gene expression profiles in PCOS ovaries to control subjects and female to male transsexuals [193]. These authors draw special attention on the differentiation promoting effect and interplay between the histon deacetylase (HDAC) function and the *PPAR-γ* gene [193]. Interestingly the antiepileptic drug valproic acid has both HDAC inhibitory activity and PPAR-*γ* agonist action that create a condition that resembles the PCOS picture during administration [194]. As a consequence, further functional studies are required to elucidate the transcriptional cascade and the events that link PPAR-*γ* function to PCOS pathogenesis.

### 4.6. Genes involved in chronic inflammation

Chronic inflammation appears to play a role in the development of insulin resistance (Figure 1) and cardiovascular disease [195] and it might be involved in the pathogenesis of PCOS [196] although not all agree [189]. Some genomic variants related to inflammation have been studied in PCOS.

#### 4.6.1. TNF-*α*

Tumor necrosis factor (TNF)-*α* is cytokine secreted by adipose tissue that plays an important role in insulin resistance [197]. The variants that are studied up to date are −308 G/A [185, 186, 198], −1196 C/T, −1125 G/C, −1031 T/C, −863 C/A, −857 C/T, −316 G/A, −238 G/A, −163 G/A [198], and −850 C/T [187]. The polymorphisms in the *TNF-α gene* do not seem to have a key role in the pathogenesis of PCOS. In a study by Escobar-Morreole et al., the carriers of −308 A alleles showed increased serum androgen and 17-hydroxyprogesterone levels before and after stimulation with the GnRH analogue leuprolide [198]. These findings may indicate that *TNF-α gene* polymorphism might be a modifying factor for phenotypic traits.

Other genes involved in chronic inflammation, such as *TNFR2* (type-2 TNF receptor) gene [188], *IL-6* [189, 199], *IL-6 signal transducer gp 130* [190], *IL-6 receptor* [190] genes have also been investigated as a candidate gene for pathogenesis of PCOS. The results of these candidate gene studies and those discussed above are summarized in Table 2.

### 4.7. Other genes

#### 4.7.1. Plasminogen activator inhibitor-1 gene

Patients with PCOS cluster cardiovascular risk factors [6]. Abnormalities in the coagulation and fibrinolytic pathways contribute to the development of CVD in PCOS [200]. Elevated plasminogen activator inhibitor-1 (PAI-1) levels are associated with increased cardiovascular risk and increased thrombogenic tendency, and in general, women with PCOS also have an increased activity of PAI-1 [6]. The 4G/5G polymorphism in the promoter region of the *PAI-1* is associated with increased plasma PAI-1 concentrations in patients with type-2 diabetes and myocardial infarction [201]. In order to understand the role of the *PAI-1* polymorphism in PCOS patients, Diamanti-Kandarakis et al. investigated this polymorphism in Greek women with PCOS and found a higher frequency of the 4G/4G and 4G/5G genotypic subtypes in PCOS compared with controls [202]. These investigators also reported that PCOS women have higher levels of PAI-1 and that the presence of the 4G allele in the *PAI-1* promoter region of the gene further increases the PAI-1 levels [202].

In addition to the genes mentioned above in detail, many different genes such as *HSD3B2* [35, 203], *17α-hydroxysteroid dehydrogenases* [35, 204], *dopamine receptor* [205, 206], *IGF* [35, 163], *aldosterone synthetase* [207], *paraoxonase* [163], *glycogen synthetase* [208], *resistin* [209], *apoprotein E* [210] have been studied up to date in a variety of PCOS populations. Similar to the studies described in detail above, either negative or controversial results have been obtained.

## 5. LIMITATIONS AND CHALLENGES FOR GENETIC STUDIES IN PCOS

PCOS is a common and complex disorder. Similar to other complex traits such as type-2 diabetes and obesity, the complexity of the underlying genetic model as well as potential gene-gene and gene-environment interactions pose a difficulty for genetic analyses. Major limitations and challenges in the genetic studies of PCOS are shown in Table 4.

### 5.1. Issues related to phenotype and genotype

There are several major limitations relevant to the phenotype and genotype studies of PCOS. Firstly, there has been an ongoing debate for the definition of and the diagnostic criteria for PCOS, and different diagnostic criteria used in different studies introduce complexity into the comparison of the results [211]. Secondly, how to assign affected status of men in PCOS families remains obscure although several clinical and biochemical characteristics have been suggested for the male phenotype including premature baldness, increased pilosity, increased DHEAS levels, exaggerated responses to GnRH or ACTH, and insulin resistance and glucose intolerance [109]. Thirdly, relatively small sample sizes of the study populations might result in statistical errors in population-based genetic studies of association for unrelated cases and controls [212].

Linkage studies run on large families spanning at least three generations including more than one affected and unaffected individuals are successful for the identification of a susceptibility locus. As infertility is one of the primary manifestations of PCOS, this is the most important fact which limits the availability of large pedigrees. In addition, nonrandom assignment of families increases the potential for selection bias. We should also note that since the mode of inheritance expressivity and penetrance is obscure, it is difficult to assess if one individual is affected or unaffected. Moreover, comparison of one pedigree to another may also harbor obstacles due that PCOS appears to be a highly heterogeneous disorder, with locus and allelic heterogeneity both between and within families likely.

### 5.2. Environmental factors and compensatory adaptation

The impact of environmental factors for the development of PCOS is currently an active area of research. One important point is that the compensatory adaptations achieved in the intrauterine life might be associated with PCOS later in life (reviewed by Escobar-Morreale [213]). There is a recent evidence from animal studies that metabolic compensatory adaptations of carbohydrate metabolism and insulin signaling acquired in the intrauterine life is carried on to the postnatal life [214]. This intrauterine compensatory adaptation might as well be implicated by epigenetic factors such as DNA methylation.

## 6. CONCLUSIONS AND FUTURE PERSPECTIVES

Based on familial, metabolic, and endocrine data, PCOS can be taken as a complex genetic trait similar to type-2 diabetes and obesity. It appears that there are multiple inherited, environmental or acquired factors that may increase the risk for developing PCOS. The efforts to identify the genes and mutations predisposing to PCOS up to date have been primarily based on association and linkage studies with functional candidate genes only a few of which exhibited significant evidence of association with PCOS. Furthermore, most of the positive findings obtained with PCOS phenotypes have not been replicated in more than one population. These results are not surprising considering that genes with such a great impact on the phenotype are not expected to be responsible for PCOS. However, it is important to emphasize that relatively small sample size of study populations is a major limitation for most of the studies.

Identification of the candidate genes and understanding of their function holds the promise for the establishment of the specific molecular basis for PCOS. More studies with larger sample sizes on different populations are needed to determine whether suggested functional candidate genes play any role at all in the etiology of PCOS. Two recent advances in genetics, namely genome-wide scan approach and the HapMap project, will serve as tools for both identification of novel loci and narrowing the known genomic regions for PCOS. The acquisition of genetic information regarding PCOS will be most useful for providing new insights into the pathophysiology of the syndrome and will eventually have a profound effect on the clinical management of the PCOS patients.

## Figures and Tables

**Table 1:** Summary of the studies of familial aggregation in PCOS.

Suggested inheritance	Diagnostic criteria	Phenotype in first-degree relatives	References

Autosomal dominant with variable penetrance	Oligomenorrhea, hirsutism, and ^(a)^PCO	*Women*: oligomenorrhea and PCO	Cooper et al. [8]

X-linked	Oligomenorrhea, hirsutism, and PCO	*Women*: hyperandrogenism and metabolic disorders	Givens et al. [9, 10]
		*Men*: oligospermia and LH hypersecretion	

Not determined	Hirsutism and/or oligomenorrhea	*Women*: infertility, oligomenorrhea, and hirsutism	Ferriman and Purdie [11]

Autosomal dominant	Menstrual dysfunction, hyperandrogenism, obesity, infertility, and PCO	*Women*: hyperandrogenic symptoms	Lunde et al. [12]
		*Men*: premature baldness and increased hairiness	

Not determined	Menstrual irregularities, hirsutism, infertility, PCO, and obesity	*Women*: PCO	Hague et al. [13]

Monogenic	PCO	*Women*: PCO	Carey et al. [14]
		*Men*: premature baldness	

Not determined	Elevated androgens, decreased SHBG, and PCO	*Men*: premature baldness, hypertriglyceridemia, and hyperinsulinemia	Norman et al. [15]

Not determined	NICHD	*Women*: Beta-cell dysfunction	Colilla et al. [16]

Monogenic	NICHD	*Women*: PCOS (NICHD), hyperandrogenemia, and insulin resistance	Legro et al. [17–19]

Not determined	NICHD	*Women*: PCOS (NICHD)	Kahsar-Miller et al. [20]

Not determined	NICHD	*Women*: PCOS (NICHD) and insulin resistance	Yildiz et al. [21]
		*Men*: insulin resistance	

^(a)^ *PCO: polycystic ovaries.*

**Table 2:** Summary of the studies of candidate genes in PCOS.

Gene	Locus/variant	Subjects/phenotypic trait	^(*a*)^Assoc.	References

*Genes involved in ovarian and adrenal steroidogenesis*				

CYP11a	(tttta)_*n*_	PCOS/	Yes	Gharani et al. [29]
		hyperandrogenemia		
		PCOS/	Yes	Wang et al. [31]
		body mass index		
		PCOS/	Yes	Diamanti-Kandarakis et al. [30]
		hyperandrogenemia		

	D15S519	PCOS/	No	Urbanek et al. [35]
	D15S520	hyperandrogenemia		

	(tttta)_*n*_	Hirsutism/	No	San Millán et al. [36]
		hyperandrogenism		

	(tttta)_*n*_	PCOS/	No	Tan et al. [33, 34]
		hyperandrogenemia		

	D15S520,1500 bp to D15S520	PCOS, ^(*b*)^PCO/	No	Gaasenbeek et al. [32]
		testosterone levels		

CYP21	Heterozygosity for CYP21 mutations	Premature pubarche/	Yes	Witchel et al. [39]
		hyperandrogenism	
		Hyperandrogenism	Yes	Witchel et al. [40]
		Hirsutism/	No	Escobar-Morreale et al. [41]
		origin of androgen excess		
		Hirsutism, PCOS/	No	Glintborg et al. [42]
		adrenal hyperresponsiveness		
		PCOS/	No	Witchel et al. [43]
		hyperandrogenemia		

CYP17	−34T/C ^(*c*)^SNP	PCOS	Yes	Carey et al. [52]
		PCOS/	Yes	Diamanti-Kandarakis et al. [53]
		hyperandrogenemia		
		PCOS/	No	Gharani et al. [54]
		hyperandrogenemia		
		PCOS/	No	Techatraisak et al. [55]
		hyperandrogenemia		
		PCOS/	No	Marszalek et al. [57]
		hormone profile		

	Mutation scanning/	Mild	No	Witchel et al. [56]
	no mutation	hyperandrogenism		

	D10S192	PCOS	No	Urbanek et al. [35]

	T/C substitution in the 5′ ^(*d*)^PR	PCOS/	No	Kahsar-Miller et al. [58]
		^(*e*)^DHEAS levels		

CYP19	CYP19	PCOS	No	Urbanek et al. [35]

	(tttta)_*n*_/D15S103	PCOS	No	Gharani et al. [29]

	Aromatase SNP_50 genotype Aromatase distal promoter region variation	PCOS symptom score testosterone concentrations	Yes	Petry et al. [74, 75]

*Genes involved in steroid hormone effects*				

AR	AR	PCOS	No	Urbanek et al. [35]

*SHBG gene*	AR (CAG)_*n*_	PCOS/infertile and	No	Mifsud et al. [83]
		fertile women		
		PCOS/infertility	Yes	Hickey et al. [84]
		PCOS/^(*f*)^BMI,	No	Jääskeläinen et al. [85]
		testosterone levels		

	(TAAAA)_*n*_	PCOS/SHBG levels	Yes	Xita et al. [90]

	Asp327Asn	Hirsutism/SHBG levels	Yes	Cousin et al. [91]

	D17S1353	PCOS	No	Urbanek et al. [35]

*Genes involved in gonadotropin action and regulation*				

*LHβ*	Trp8Arg; Ilg15Thr	PCOS/higher frequency of	Yes	Rajkhowa et al. [98]
		the SNPs in obese PCOS	
		PCOS/lower frequency of	Yes	Tapanainen et al. [99]
		the SNPs in obese PCOS		
		Menstrual disorders	No	Ramanujam et al. [100]

	Ser102 Gly	Menstrual disorders	Yes	Ramanujam et al. [100]

	Trp8Arg; Ilg15Thr	PCOS	No	Elter et al. [101]

	Several SNPs	Ovulatory disorders	Yes	Takahashi et al. [103]
		including PCOS		

*Follistatin gene*	D5S474	PCOS	Yes	Urbanek et al. [35]
	D5S623			
	D5S822			

	Mutation scanning/	PCOS	No	Urbanek et al. [110]
	variants at 17 sites			

	Mutation scanning/	PCOS	No	Liao et al. [111]
	no mutation			

	Mutation scanning/	PCOS/hormone profile	No	Calvo et al. [112]
	no mutation except			
	G951A (silent mutation)			

*Genes involved in insulin action and secretion*				

*INS*	*INS VNRT*	PCOS/premature male	Yes	Waterworth et al. [123]
		pattern baldness		
		PCOS	Yes	Eaves et al. [124]
		PCOS/insulin resistance	Yes	Michelmore et al. [125]
		and hyperandrogenemia		
		PCOS/	No	Urbanek et al. [35]
		hyperandrogenemia		
		Hyperandrogenemia	No	Calvo et al. [126]
		PCOS/insulin	No	Vankova et al. [127]
		secretion and action		
		PCOS; PCO;	No	Powell et al. [129]
		testosterone level		

*INSR*	Mutation scanning	PCOS/insulin resistance	No	Talbot et al. [133]

	D19S884	PCOS	Border	Urbanek et al. [35]
		PCOS	Yes	Tucci et al. [134]
		PCOS	No	Villuendas et al. [135]
		PCOS	Yes	Urbanek et al. [136]

	C10923T	PCOS/lean subjects	Yes	Siegal et al. [137]

*IRS*	*IRS1*	PCOS	No	Urbanek et al. [35]

	Gly972Arg (*IRS1*)	PCOS	Yes	Sir-Petermann et al. [142]

	Gly972Arg (*IRS1*)	PCOS/insulin resistance	Yes	El-Mkadem et al. [143]
	Gly1057Asp(*IRS2*)			

*IRS*	Gly972Arg (*IRS1*)	PCOS/insulin and glucose levels	No	Ehrmann et al. [144]

	Gly972Arg (*IRS1*)	PCOS/glucose homeostasis	No	Villuendas et al. [145]
	Gly1057Asp(*IRS2*)			

	Gly972Arg (*IRS1*)	PCOS/insulin resistance, and obesity	Yes	Dilek et al. [146]
		PCOS/insulin resistance, hormone profile, and metformin effects	Yes	Ertunc et al. [147]

*CAPN10*	UCSNP-19, -43, and -63	PCOS/insulin levels	Yes	Ehrmann et al. [152]

	UCSNP-19, -43, -44, and -63	PCOS	Yes	Gonzales et al. [153, 154]
		PCOS/insulin levels	No	Haddad et al. [155]

	UCSNP-43	Hirsutism/hirsutism score	Yes	Escobar-Morreale et al. [156]

	UCSNP-44	Hirsutism/PCOS, idiopathic hirsutism, and hyperandrogenism	No	Escobar-Morreale et al. [156]

	UCSNP-45	Hirsutism/idiopathic hirsutism	Yes	Escobar-Morreale et al. [156]

*Genes involved in energy homeostasis*				

*Leptin gene & Leptin receptor*	Mutation scanning of the leptin gene; polymorphism in leptin receptor gene	PCOS/obesity	No	Oksanen et al. [158]

*Adiponectin gene*	T45G in exon 2	PCOS	No	San Millán et al. [163]
	G276T in intron 2			

	T45G in exon 2	PCOS	No	Panidis et al. [164]

	T45G in exon 2	PCOS	No	Xita et al. [165]
	G276T in intron 2	PCOS	No	Escobar-Morreale et al. [166]

*Genes involved in chronic inflammation*				

*TNF-α*	−308 G/A	PCOS	No	Milner et al. [185]
		PCOS	No	Mao et al. [186]
		PCOS	No	Korhonen et al. [187]

*TNFR2 gene*	Met196Arg	PCOS/	Yes	Peral et al. [188]
		hyperandrogenism	

*IL-6*	−174 G/C	PCOS	Yes	Mohlig et al. [189]

*IL-6 signal*	Gly148Arg	PCOS/	Yes	Escobar-Morreale et al. [190]
*transducer gp 130*		hyperandrogenism		

*IL-6 receptor-α*	CA repeats	PCOS/obesity, and Hyperandrogenism	Yes	Escobar-Morreale et al. [190]

^(*a*)^Assoc.: association.

^(*b*)^PCO: polycystic ovaries.

^(*c*)^SNP: single nucleotide polymorphism.

^(*d*)^PR: promoter region.

^(*e*)^DHEAS: dehydroepiandrosterone sulfate.

^(*f*)^BMI: body mass index.

**Table 3:** Genes involved in energy homeostasis: *PPAR-γ*.

Gene	Locus/variant	Subjects/phenotypic trait	^(*a*)^Assoc.	References

*PPAR-γ Gene*	D3S1263	PCOS	No	Urbanek et al. [35]

	Pro12Ala	PCOS/reduced insulin resistance	Yes	Hara et al. [176]
		PCOS/lower hirsutism scores,	Yes	Hahn et al. [177]
		increased insulin sensitivity		
		Reduced insulin resistance	^(*b*)^ Yes	Tok et al. [178]
		PCOS	Yes	Korhoren et al. [179]
		PCOS	No	San Millán et al. [163]
		PCOS	No	Orio et al. [182]

		PCOS/clinical and hormonal	Yes	Yilmaz et al. [181]
		characteristics		

	CAC478CAT	PCOS/obesity, leptin levels	Yes	Orio et al. [182]

*PPAR-γ* &	Pro12Ala &	PCOS	No	Wang et al. [183]
*PGC-1α*	Gly482Ser			

^(*a*)^Assoc.: association. ^(*b*)^ *No statistical significance*.

**Table 4:** Major limitations of genetic studies in PCOS.

Lack of universally accepted diagnostic criteria and definition	• NICHD criteria
	• Ultrasonographic criteria
	• Rotterdam criteria

Male phenotype?	• Premature baldness
	• Increased pilosity
	• Increased DHEAS levels?
	• Exaggerated responses to GnRH and ACTH
	• Insulin resistance, glucose intolerance

Relatively small sample size of the study populations	• Potential statistical error

Affected reproduction	• Difficulty in studying more than one generation

Nonrandom ascertainment of families	• Bias?

Obscurity in the mode of inheritance	• Autosomal Dominant [8, 12]
	• Monogenic [14, 17]
	• X-Linked [9, 10]

Variable penetrance and expressivity	• Difficulty in assignment of the phenotype
	(affected versus unaffected)

Locus heterogeneity	• Summarized in Section 4.

Environmental interactions	• Compensatory adaptation?
